# Unusual Manifestation of Bilateral Intermediate Uveitis Caused by Ocular Cytomegalovirus (CMV) in Immunocompetent Individual

**DOI:** 10.7759/cureus.77737

**Published:** 2025-01-20

**Authors:** Izzati Othman, Evelyn Tai Li Min, Norlelawati Abu

**Affiliations:** 1 Department of Ophthalmology and Visual Science, Universiti Sains Malaysia, Kubang Kerian, MYS; 2 Department of Ophthalmology, Hospital Tuanku Ja’afar, Seremban, MYS

**Keywords:** cmv immunocompetent, cmv uveitis, cytomegalovirus (cmv), intermediate uveitis, ocular cmv

## Abstract

Anterior uveitis frequently occurs in ocular cytomegalovirus (CMV) infection among immunocompetent individuals. Here, we report a rare instance of bilateral isolated intermediate uveitis without retinitis or retinal vasculitis in an immunocompetent individual, later confirmed to be an ocular CMV infection. He was treated with systemic ganciclovir and topical corticosteroid. His symptoms and ocular signs have significantly improved. This case highlights the diverse ocular manifestations seen in ocular CMV infection among immunocompetent individuals.

## Introduction

Uveitis is an eye disease responsible for 2.8% to 10% of visual impairment worldwide [1]. Differential diagnosis is often challenging and typically relies on clinical presentation, laboratory findings, and the treatment course. The growing availability of molecular techniques, such as polymerase chain reaction (PCR), has significantly improved the diagnosis of infectious uveitis, especially herpetic uveitis.

The spectrum of cytomegalovirus (CMV) uveitis reported consists of anterior uveitis (AU) and posterior uveitis (PU). CMV anterior uveitis (CMV AU) is the most frequent ocular manifestation of CMV infection in immunocompetent individuals. Recurrence and chronicity are common and can result in irreversible vision loss [2]. Conversely, CMV posterior uveitis primarily occurs in immunocompromised patients, although several cases have been reported in immunocompetent individuals [3]. We experienced an immune-competent individual who developed bilateral intermediate uveitis (IU) due to CMV infection which is rare and atypically seen. Informed consent was taken from the patient for us to document this case report.

## Case presentation

**Figure 1:**
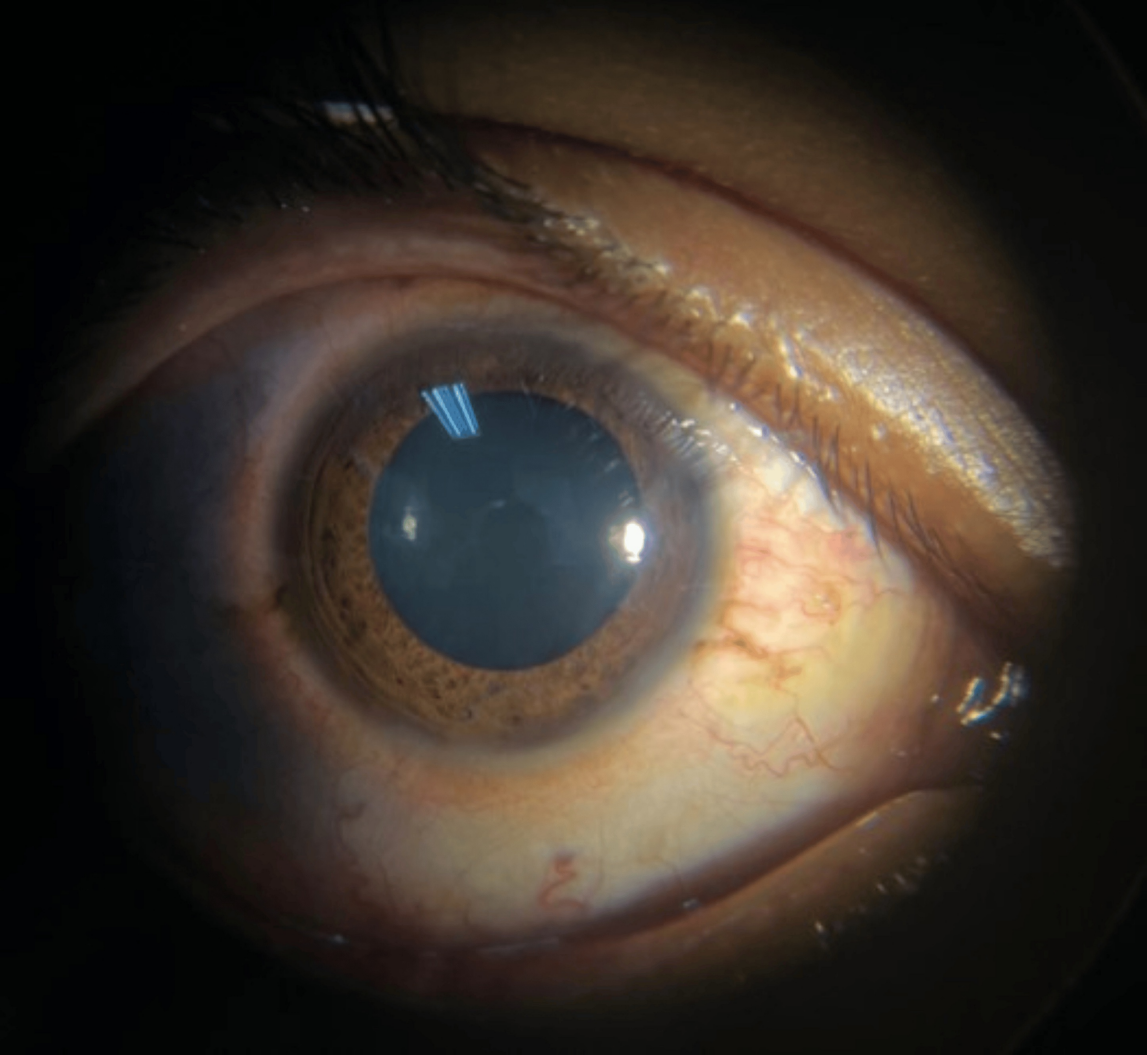
Right anterior segment photo showed pharmacologically well dilated pupil with absence of peripheral synechiae even with the presence of anterior chamber cells of 3+ (cells not well visualized in this photo).

A 23-year-old healthy gentleman presented with a six-week duration of painless redness in his left eye, accompanied by a gradual reduction in vision. He had a history of low-grade fever lasting for three days, occurring two weeks prior to the onset of ocular symptoms. He had no constitutional symptoms and denied high-risk behavior. Upon examination, right visual acuity was 6/9. The anterior segment of the right eye showed 3+ cells with a 3 mm round and reactive pupil (Figure 1).

**Figure 2:**
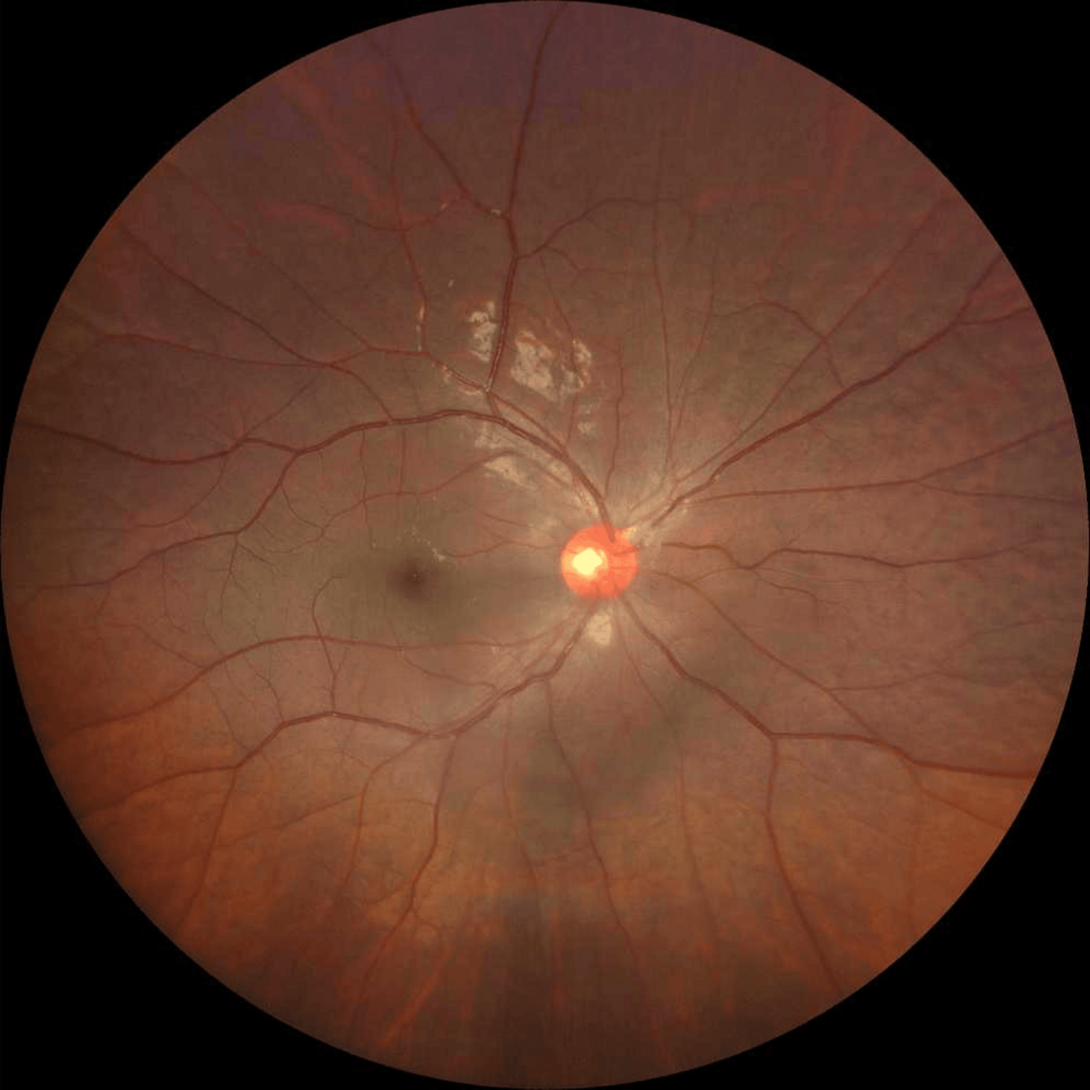
Right fundus showed minimal vitritis with absence of retinitis, choroiditis or vasculitis

Fundus examination revealed mild vitritis without evidence of retinitis, choroiditis or vasculitis (Figure 2).

**Figure 3:**
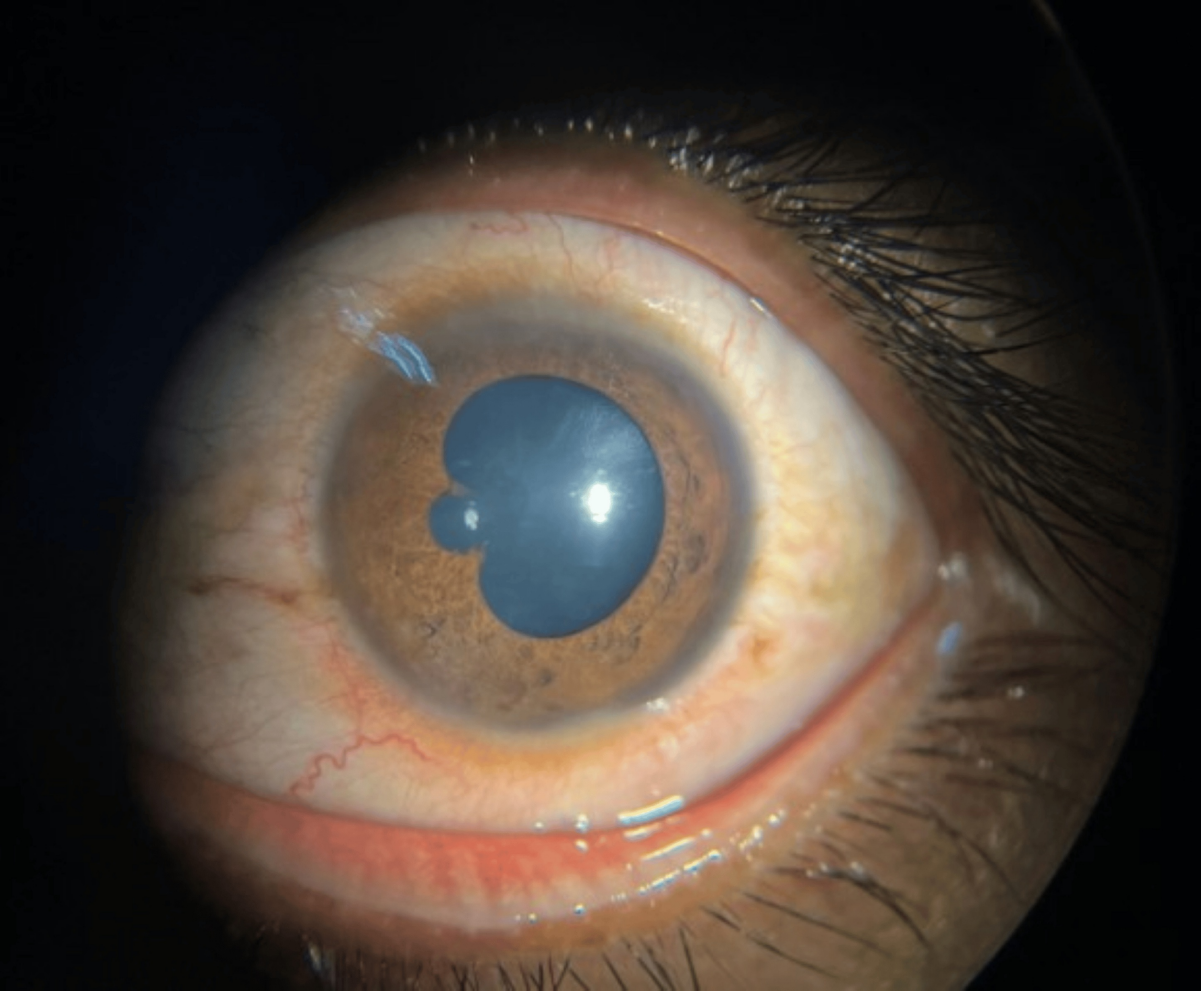
Left irregular pupil shaped due to peripheral synechiae located at 8 and 10 o’clock.

The left visual acuity was 6/18. The left conjunctiva was injected however the cornea was clear with fine keratic precipitates on the endothelium. Anterior chamber cells were observed as 4+ with a fibrin clump from 9-11 o’clock. The irregular pupil was attributed to the presence of posterior synechiae at 8 and 10 o’clock (Figure 3).

**Figure 4:**
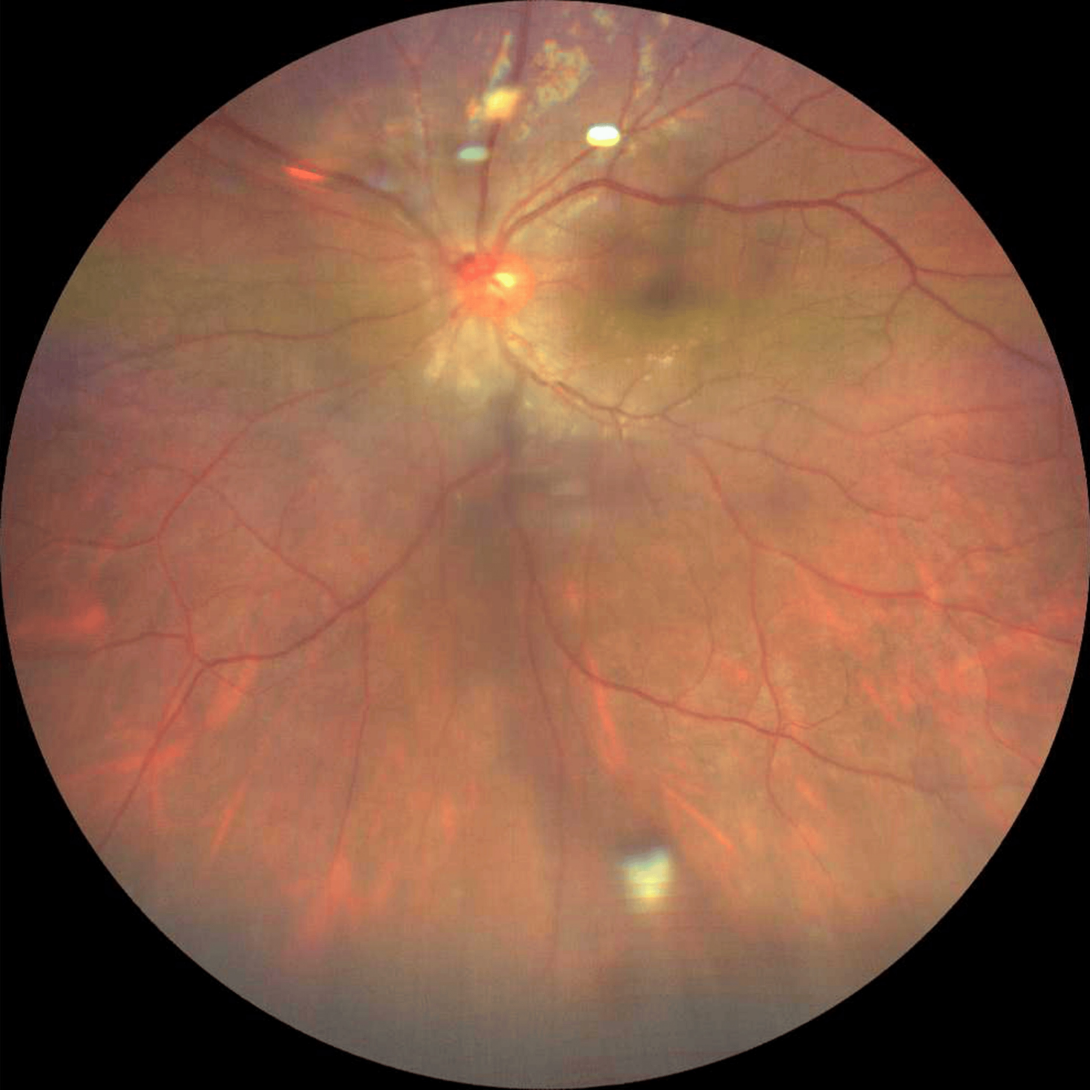
Fundus photo of the left eye shows vitritis clump located at inferotemporal quadrant of the peripheral retina.

Fundus examination showed vitritis, optic disc hyperemia and absence of retinitis, choroiditis and vasculitis (Figure 4).

Bilateral intraocular pressure was 14 mmHg. Systemic examination was unremarkable. Chest X-ray and Mantoux test were negative. Autoimmune disease workups such as rheumatoid factor, anti-ds-DNA, and antinuclear antibody (ANA) to rule out autoimmune disease were also negative. A complete blood count with differentials together with infectious screenings yielded negative results except for a significantly elevated level of Cytomegalovirus Immunoglobulin G (CMV IgG) at 1927 IU/ml. The diagnosis of ocular CMV was confirmed with a positive Polymerase Chain Reaction test (PCR) of the left eye's aqueous humor.

He was diagnosed with bilateral intermediate uveitis due to CMV ocular infection. Intravenous ganciclovir and topical steroids were administered and his clinical condition was improving with the treatment.

## Discussion

Herpetic uveitis is a form of infectious uveitis caused by Herpesviridae, a large family of double-stranded DNA viruses [1]. This family includes over a hundred viruses, some of which are well-known pathogens, such as herpes simplex virus (HSV), varicella-zoster virus (VZV), and cytomegalovirus (CMV) [1]. CMV AU is the most common ocular manifestation of CMV infection in immunocompetent individuals, often characterized by recurrence and chronicity, which can result in irreversible vision loss [1,2]. In contrast, CMV PU typically occurs in immunocompromised patients, although there have been several documented cases in immunocompetent patients [3]. To date, only one case of CMV IU in immunocompetent individuals has been reported, making it a rarity [4].

CMV IU presented with mild conjunctival injection, moderate anterior chamber inflammation, and diffuse, fine, stellate keratic precipitates (KPs) evenly distributed over the endothelium, resembling Fuchs Uveitis Syndrome (FUS). It may also be associated with high intraocular pressure, as reported by Amin et al. [4,5].

PCR's sensitivity to detect trace amounts of DNA ensures a high positive detection rate during the early stages of CMV infection [6]. A qualitative multiplex PCR can be used for virus screening, while real-time PCR aids in identifying and quantifying viruses, making it a standard tool for diagnosis and assessing disease severity [7]. Additionally, real-time PCR can be utilized to monitor treatment effectiveness [8].

Following the prompt diagnosis of CMV infection based on clinical symptoms and aqueous humor analysis, early antiviral treatment is essential to prevent complications such as glaucoma [9], endothelial dysfunction, permanent corneal damage, and retinal detachment. Ganciclovir and valganciclovir inhibit CMV DNA polymerase UL54 by competitively blocking viral DNA synthesis [10]. Ganciclovir is commonly used for both systemic (intravenous) and topical (intravitreal injection, eye drops, or gel) antiviral therapy, while valganciclovir is primarily used for systemic (oral) therapy. Systemic treatment is generally more effective at controlling inflammation than topical treatment [11], likely due to higher therapeutic concentrations of ganciclovir being maintained in the aqueous humor. Both oral and intravenous therapies are effective at reducing inflammation and lowering intraocular pressure. While side effects in immunocompetent individuals are typically mild, regular blood tests are necessary during systemic therapy to monitor complete blood counts and renal function.

## Conclusions

Although CMV AU is the most common ocular manifestation of CMV infection in immunocompetent individuals, it can also manifest as CMV IU. The presentation of CMV-related IU in these patients can vary, highlighting the need for additional documentation and reports for future reference.

If left untreated, CMV IU may progress to complications such as iris atrophy, glaucoma, and retinal detachment, making early diagnosis and treatment critical for a favorable outcome. Accurate diagnosis is achieved through RT-PCR analysis of aqueous humor. Antiviral treatment options include intravenous and topical ganciclovir, as well as oral valganciclovir.

## References

[1] Miserocchi E, Fogliato G, Modorati G, Bandello F (2013). Review on the worldwide epidemiology of uveitis. Eur J Ophthalmol.

[2] Chan NS, Chee SP, Caspers L, Bodaghi B (2018). Clinical features of CMV-associated anterior uveitis. Ocul Immunol Inflamm.

[3] Tabatabaei SA, Cheraqpour K, Pour EK, Bohrani Sefidan B (2020). Long-term prophylaxis in an immunocompetent patient with Cytomegalovirus retinitis: a case report and review of literature. J Ophthalmic Inflamm Infect.

[4] Amin RM, Ostheimer T, Butler NJ (2015). Intermediate uveitis: an unusual presentation of cytomegalovirus intraocular infection in an immunocompetent patient. BMJ Case Rep.

[5] Chee SP, Bacsal K, Jap A, Se-Thoe SY, Cheng CL, Tan BH (2008). Clinical features of cytomegalovirus anterior uveitis in immunocompetent patients. Am J Ophthalmol.

[6] Mochizuki M, Sugita S, Kamoi K, Takase H (2017). A new era of uveitis: impact of polymerase chain reaction in intraocular inflammatory diseases. Jpn J Ophthalmol.

[7] Kandori M, Miyazaki D, Yakura K, Komatsu N, Touge C, Ishikura R, Inoue Y (2013). Relationship between the number of cytomegalovirus in anterior chamber and severity of anterior segment inflammation. Jpn J Ophthalmol.

[8] Touhami S, Qu L, Angi M (2018). Cytomegalovirus anterior uveitis: clinical characteristics and long-term outcomes in a French series. Am J Ophthalmol.

[9] Crumpacker CS (1996). Ganciclovir. N Engl J Med.

[10] Chee SP, Jap A (2010). Cytomegalovirus anterior uveitis: outcome of treatment. Br J Ophthalmol.

[11] Sobolewska B, Deuter C, Doycheva D, Zierhut M (2014). Long-term oral therapy with valganciclovir in patients with Posner-Schlossman syndrome. Graefes Arch Clin Exp Ophthalmol.

